# A Comparison between the Online Prognostic Tool PREDICT and myBeST for Women with Breast Cancer in Malaysia

**DOI:** 10.3390/cancers15072064

**Published:** 2023-03-30

**Authors:** Mohd Nasrullah Nik Ab Kadir, Suhaily Mohd Hairon, Imi Sairi Ab Hadi, Siti Norbayah Yusof, Siti Maryam Muhamat, Najib Majdi Yaacob

**Affiliations:** 1 Department of Community Medicine, School of Medical Sciences, Universiti Sains Malaysia, Kubang Kerian 16150, Kelantan, Malaysia; 2 Breast and Endocrine Surgery Unit, Department of Surgery, Hospital Raja Perempuan Zainab II, Ministry of Health Malaysia, Kota Bharu 15586, Kelantan, Malaysia; 3 Malaysian National Cancer Registry Department, National Cancer Institute, Ministry of Health Malaysia, Putrajaya 62250, Federal Territory of Putrajaya, Malaysia; 4 Biostatistics and Research Methodology Unit, School of Medical Sciences, Universiti Sains Malaysia, Kubang Kerian 16150, Kelantan, Malaysia

**Keywords:** breast neoplasm, prognosis, women, Malaysia, online prognostic tool

## Abstract

**Simple Summary:**

Prognostic tools are valuable for risk communication. The popular PREDICT breast cancer tool was less accurate in predicting survival among Malaysian women. A new web-based prognostic tool, the Malaysian Breast cancer Survival prognostic Tool (myBeST), was developed to address the limitations. It was based on the model’s algorithm derived from local patients’ experiences. In this study, we compare both tools’ prediction performance among women with breast cancer in Malaysia involving a cohort of 532 patients. Both models are satisfactory, but myBeST exceeds PREDICT performances in discriminant properties. Hence, the myBeST model is more applicable to our population to convey survival estimation and manage patient expectations.

**Abstract:**

The PREDICT breast cancer is a well-known online calculator to estimate survival probability. We developed a new prognostic model, myBeST, due to the PREDICT tool’s limitations when applied to our patients. This study aims to compare the performance of the two models for women with breast cancer in Malaysia. A total of 532 stage I to III patient records who underwent surgical treatment were analysed. They were diagnosed between 2012 and 2016 in seven centres. We obtained baseline predictors and survival outcomes by reviewing patients’ medical records. We compare PREDICT and myBeST tools’ discriminant performance using receiver-operating characteristic (ROC) analysis. The five-year observed survival was 80.3% (95% CI: 77.0, 83.7). For this cohort, the median five-year survival probabilities estimated by PREDICT and myBeST were 85.8% and 82.6%, respectively. The area under the ROC curve for five-year survival by myBeST was 0.78 (95% CI: 0.73, 0.82) and for PREDICT was 0.75 (95% CI: 0.70, 0.80). Both tools show good performance, with myBeST marginally outperforms PREDICT discriminant performance. Thus, the new prognostic model is perhaps more suitable for women with breast cancer in Malaysia.

## 1. Introduction

Breast neoplasm is a public health priority contributing substantially to global mortality and disability-adjusted life-years (DALYs). Nearly all countries and territories rank breast cancer as the leading type of female cancer [1,2]. The incidence rates of breast cancer increase, albeit reducing trend of mortality rates and DALYs [3]. Similarly, women with breast cancer in Malaysia contributed 33.9% of new cancer cases diagnosed between 2012 and 2016, leaving colorectal cancer (10.7%) far behind as the second most commonest female cancer [4]. Individuals diagnosed with breast cancer face enormous challenges in terms of financial cost [5,6], adherence to multimodal intervention and long-term cancer surveillance [7,8].

Prognostic tools were developed to improve treatment adherence and patient’s expectation of the disease outcomes, especially during the survivorship period. The frequently validated and helpful models are the Nottingham Prognostic Index (NPI), Adjuvant! Online, Cancer Math and PREDICT breast cancer (PREDICT) [9,10,11,12,13]. These tools predicted the survival probabilities for each patient according to her unique clinical and pathological characteristics as well as the treatment received. PREDICT was the latest tool to be developed and underwent regular updates [14,15]. Among these tools, PREDICT performed best, as found by validation studies conducted among women with breast cancer in Malaysia [16,17,18].

PREDICT breast cancer is the most frequently used tool to aid clinical decision-making. It was initially developed in 2010 based on women with breast cancer diagnosed between 1989 and 2003 [14]. The tool underwent several upgrades to include new prognostic markers such as human epidermal growth factor receptor 2 (HER2) and Ki-67, as well as the addition of new intervention strategies such as trastuzumab, bisphosphonate and extended hormone treatment. The latest version was published in 2017 [15]. Several validation studies showed acceptable prediction accuracy to use the tool in clinical practice [15,18,19,20]. Both the American Joint Commission on Cancer (AJCC) and UK National Institute for Health and Clinical Excellence (NICE) endorsed the tool [21].

The PREDICT tool had several limitations in predicting five-year survival among women with breast cancer in Malaysia. It was less accurate among patients younger than 40, of the Malay ethnic group, those with ER-negative tumours and those receiving neoadjuvant chemotherapy [18]. However, the validation dataset was limited to those attending one urban academic centre that might differ from the rest of Malaysian breast cancer patients’ experiences. In addition, it used the earlier version of PREDICT tool [18].

Considering the limitations of the previous Western-centric online tool, we developed a new prognostic tool known as myBeST, the Malaysian Breast-cancer-Survival prognostic Tool. The tool’s model algorithm was derived from multivariable Cox proportional hazard regression analysis and found to have robust calibration and discriminant performance (area under the receiver operating characteristics curve, AUC: 0.891) [22]. In addition, the predictor includes local ethnic groups, as the Asian ethnic group and particularly our population had a markedly higher mortality risk due to background genetic and lifestyle risk on top of unfavourable socio-economic and cultural-related health determinants [23,24,25,26]. The tool was deployed in a web-based format and can be accessed via http://mybestpredict.com/ (accessed on 20 February 2023) [27].

Thus, our current analysis aimed to compare the performance of locally adapted myBeST and western-centric PREDICT tools among women with breast cancer in Malaysia. The PREDICT model was chosen due to its superior performance compared to other tools (i.e., Adjuvant! Online and CancerMath) [16,17] and could predict five-year survival probability, which was the interest of this study.

## 2. Materials and Methods

### 2.1. Data Source and Study Design

We described the detailed study design and data sources in our previous paper [22], which primarily aimed to describe the development of new predictive models for survival. In summary, the sampled cohort involved women with breast cancer in Malaysia diagnosed between 1 January 2012 and 31 December 2016 that were followed for their survival outcome until 31 December 2021.

**Figure 1:**
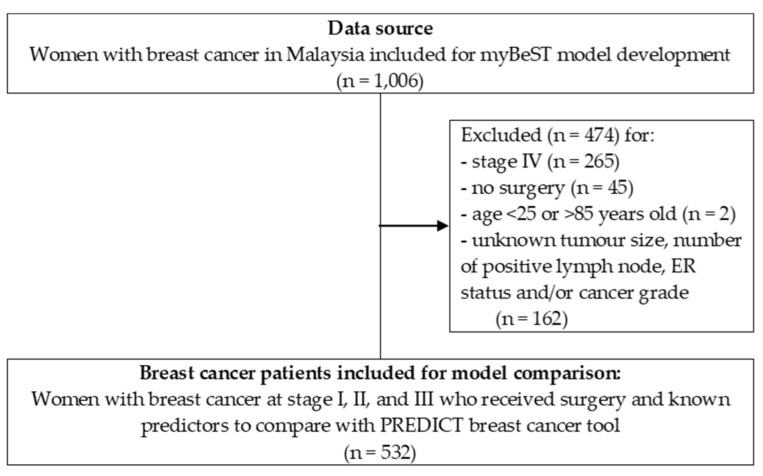
Study flow diagram of the cohort included for this analysis.

To compare with the PREDICT breast cancer, we analysed a subset of the cohort who underwent early definitive surgical intervention and were diagnosed at stage I to III according to the American Joint Committee on Cancer Staging Manual seventh edition. We included those aged between 25 and 85 and exclude those without tumour size information, the number of positive axillary lymph nodes, ER status, and cancer grade. The final dataset comprised 532 patients. The study flow diagram is as in Figure 1.

### 2.2. Analysis Method

Descriptive statistics were used to summarise the patients’ sociodemographic and clinical characteristics. The frequency and percentage of categorical data were presented. The mean/standard deviation (SD) or median/interquartile range (IQR) of numerical data was presented according to data distribution.

For PREDICT, we calculated the predicted five-year survival probability using the R package “nhspredict”, which corresponds to the PREDICT model version 2.1. Each patient’s baseline predictors were input as recorded for variables including age at diagnosis (between 25 and 85), ER status (positive or negative), HER2 status (positive, negative, or unknown), tumour grade (1, 2, or 3), invasive tumour size (in mm) and the number of positive axillary lymph nodes. The data were entered as unknown for postmenopausal status, Ki-67, and detection method. Those who received chemotherapy were input as second-generation chemotherapy. These variables were not routinely documented in the patient’s medical records, and most patients who received chemotherapy were of second-generation chemotherapy [18]. Endocrine, trastuzumab and bisphosphonates therapy’s effects were not taken into account in this study. These three predictors were entered as “No”. We followed the method used by previous comparison and validation studies [18,19,28].

For the myBeST tool, we obtained the predicted five-year survival probability by fitting the previously developed Cox proportional hazard regression model [22]. The predictors included in the model include age at diagnosis, ethnicity (Malay, Chinese, Indian or Others), marital status (married or not married), histological type (ductal carcinoma, lobular carcinoma or others), tumour grade (1, 2 or 3), ER and PR status (both ER and PR positive, either ER or PR positive, or both ER and PR negative), HER2 status (positive, negative, or unknown), tumour stage (T1, T2, T3 or T4), nodal stage (N0, N1, N2 or N3), chemotherapy (yes or no) and radiotherapy (yes or no). For this analysis, all patients received surgical treatment with no distant metastasis. The myBeST model’s algorithm did not include endocrine and anti-HER2 therapy as predictors [22,27].

The Kaplan–Maier method was employed to determine the five-year overall survival for this cohort. The survival time for each patient referred to the duration in years between the date of confirmed histological diagnosis and the date of the observed event (or the last follow-up date for censored observation). In addition, we presented the distribution and median values of survival probability for PREDICT and myBeST tools for comparison. The median values were presented to represent the centre of the predicted probabilities. This was due to the skewness of the distribution.

We measured the models’ discriminant performance using receiving operator characteristic (ROC) analysis. The area under the receiver operator (AUC) curve is the most commonly used method to assess discrimination accuracy [9,29]. An AUC of 0.5 indicated no discriminative performance (i.e., the ability to discern who survives or dies after five years of diagnosis), whereas an AUC of 1.0 implied perfect discrimination. A higher value indicated a better performance. This analysis was also used in other comparison and validation studies related to the PREDICT tool [18,28,30,31]. All analyses were conducted using R software version 4.1.3 (R Core Team: Vienna, Austria, 2020).

### 2.3. Ethics Statement

The Medical Research and Ethics Committee, Ministry of Health Malaysia (NMRR-21-37-57989 (IIR)) and the Human Research and Ethics Committee, Universiti Sains Malaysia (USM/JEPeM/21010112), granted us ethical approval. The permission to use non-identifying patient records was obtained from the data custodian, the directors of the participating centres, Ministry of Health Malaysia. We ensured the confidentiality of the patient’s data. The analysis was conducted in a manner that the subject could not be identified. The data were only accessible to the members of the research team. These data were used under agreement for the current study and are not publicly available without the express permission of the Director General of the Malaysian Ministry of Health.

## 3. Results

### 3.1. Patients’ Profile

A total of 532 women with breast cancer were included for analysis. The mean (SD) age at diagnosis was 52.1 (10.7) years. Most of the patients were Malay (58.1%), married (82.7%), had ductal carcinoma (89.1%), ER and PR positive (59.6%), HER2 negative (62.4%), at T2-(51.9%), had received chemotherapy (70.9%) and had received radiotherapy (64.7%). The median tumour size and the number of positive nodes at presentation were 29.0 mm (IQR: 20.0–45.0) and 1.0 (IQR: 0–2.0), respectively. The median follow-up time was 6.1 years (IQR: 5.2–7.5). Within five years since diagnosis, 105 (19.7%) patients died. Detailed descriptive statistics are presented in Table 1.

### 3.2. Five-Year Observed Survival and Predicted Survival Probability

**Figure 2:**
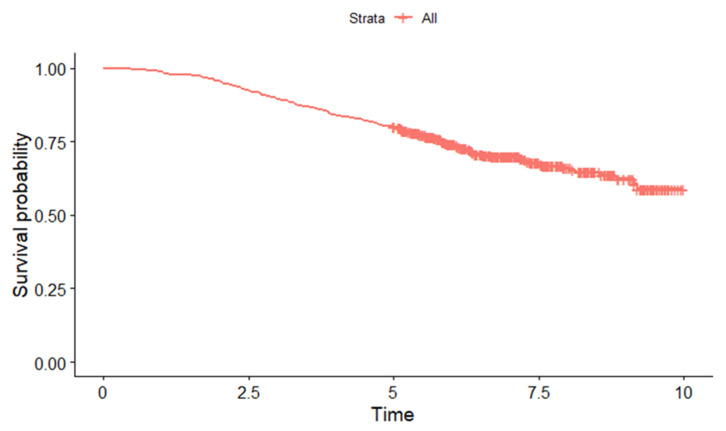
Kaplan–Maier curve for survival estimate among women with breast cancer in Malaysia included for analysis.

The five-year overall survival for all patients was 80.3% (95% CI: 77.0, 83.7). The Kaplan–Maier survival curve for the patients included for analysis is illustrated in Figure 2.

**Figure 3:**
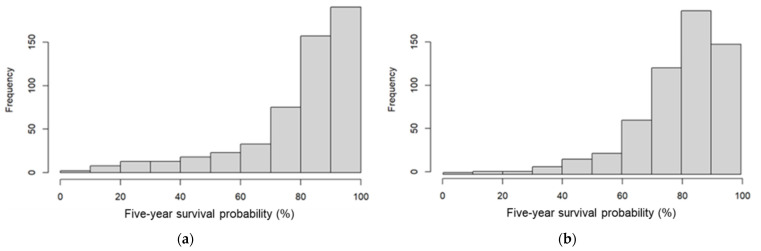
Distribution of predicted five-year survival probability by (**a**) PREDICT and (**b**) myBeST tool.

Figure 3 shows the distribution of the predicted five-year survival probabilities calculated by myBeST and PREDICT tool among women with breast cancer in this cohort. The median (IQR) survival probabilities for PREDICT and myBeST were 85.8% (73.8–93.0%) and 82.6% (71.8–90.1%), respectively.

### 3.3. Performance of PREDICT and myBeST

**Figure 4:**
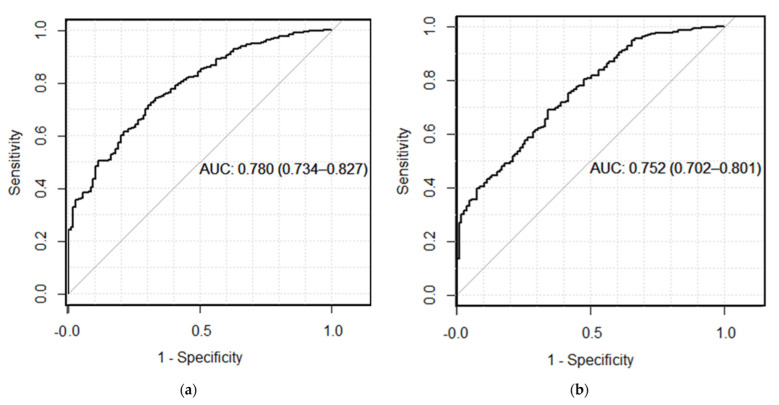
ROC curve for five-year overall breast cancer survival: (**a**) AUC for five-year survival by myBeST; (**b**) AUC for five-year survival by PREDICT.

The area under the ROC curve for five-year survival by myBeST was 0.78 (95% CI: 0.73, 0.82) and for PREDICT was 0.75 (95% CI: 0.70, 0.80). Both models had good discrimination performance by ROC analysis (Figure 4).

## 4. Discussion

Individualised breast cancer survival is important for women with breast cancer for treatment choice, adherence, and expectations of disease outcome in the survivorship period. This study compares two tools for predicting breast cancer survival probability using a cohort of Malaysian breast cancer patients. Our analysis showed that both models performed well with comparable performance in discriminating between five-year survivors and non-survivors.

Our findings were similar to the previous PREDICT validation study conducted in Malaysia. The study found no significant difference between the observed and model-predicted survival except in patients who received neoadjuvant treatment and were less than 40 years at the time of diagnosis [18]. The AUC of the study was 0.78 (95% CI: 0.74, 0.81). The study, however, enrolled patients in a single centre within an urban area with a sizeable ethnic Chinese population known to have a better prognosis and predominantly presented at an earlier stage of diagnosis [18,26,32,33]. In addition, it used the previous version of the PREDICT model. The PREDICT tool was later re-fitted into the latest version to improve the accuracy and was used to compare with myBeST in this study [14,15].

A validation study among Japanese breast cancer patients using the recent version of the PREDICT tool showed accurate prediction (AUC: 0.71, 95% CI: 0.60, 0.81), except among those aged 65 years old and above [30]. In contrast, a multicentre study among Thai patients found that the PREDICT model provided inaccurate survival prediction. The model underestimated the survival in all patients and in the subgroup analysis [28]. It could be due to the inclusion of fewer patients with unfavourable prognoses and the non-inclusion of predictors such as the progesterone receptor and Ki-67 [28]. Another study comparing a new model developed from Thai patients with the PREDICT tool found the PREDICT tool to be imprecise in survival prediction. The PREDICT model overestimates and underestimates survival in several prognostic groups [34,35]. The study attributed the findings to relatively younger age, larger tumour size, a greater number of positive nodes and a lower proportion of ER-positive tumour among Thai patients, compared to the cohort in which the PREDICT tool was derived [34].

The application of prognostic tools based on the western population in Korea echoed similar imprecise findings [36,37]. Women with breast cancer in Asia had lower survival that could be attributed to their background cardiovascular risk, comorbidities and post-diagnostic unhealthy lifestyle [18,38,39]. A model that includes these variables among Asian patients could potentially provide a more precise estimation. For example, an integrative prognostic model that includes clinical variables and modifiable risk factors was found to be superior to the PREDICT model [40].

Several reasons could have influenced our findings. The PREDICT and myBeST models’ algorithms were based on Cox PH analysis, albeit with distinct algorithms to predict survival probabilities. The PREDICT model constrains the prediction of survival based on clinical trials’ treatment effects [15], whereas the myBeST model adjusted the prediction to the treatment received by the patients. The myBeST model’s algorithm was not intended to demonstrate the treatment effects but to offer survival probability information depending on the clinical parameters and potential treatment received.

The PREDICT tool was derived from patients’ case mix with favourable outcomes. Their patients had a substantially higher portion of patients diagnosed at T1-stage (PREDICT: 51.5% vs. myBeST: 25.0%) and N0-stage (PREDICT: 62.0% vs. myBeST: 49.1%) [14,15]. Despite the inclusion of unfavourable patients’ case mix, myBeST model marginally outperformed PREDICT discriminant performance.

In addition, each tool was developed for a different reason. The PREDICT tool primarily aimed to aid adjuvant treatment decision-making based on survival outcomes that only include patients with early breast cancer cases and who underwent early definitive surgery [14,15]. The tool includes novel biomarkers as predictors, such as Ki-67, and additional treatment options, such as trastuzumab, bisphosphonate, and hormone treatments. The PREDICT model also includes the mode of detection as one of the important predictors as a diagnosis by a screening method could contribute to the bias in the survival estimation [14,15,41]. Specifying the predictors in our sample might improve the prediction.

Meanwhile, the myBeST tool was primarily developed to convey survival estimates that include all patients, including those with metastasis and not receiving treatment [27]. The model consists of all 13 predictors deemed crucial in predicting survival and routinely documented in the medical records. Selecting all variables for the modelling approach was intended to prevent data-driven variable selection. Crucially, the model’s algorithm incorporated local ethnic groups as one of the predictors that set it apart from other western-centric prognostic tools. The locally adapted measure was to reflect the differential survival outcome experienced between these groups [22,32].

Our study’s strength is that we used a relatively recent cohort of newly diagnosed breast cancer patients in Malaysia. The survival status was verified almost completely as the data were linked to the national mortality registry. Furthermore, we sampled women with breast cancer using a population-based cancer registry database as a sampling frame. They were from multiple centres across different regions in the country to better represent the heterogeneous case mix in our population.

A potential limitation of our study is the use of retrospective observational record review data with several predictors that are missing and not collected that were coded as unknown. These predictors include postmenopausal status, Ki-67, and the detection method. The PREDICT tool allows for unknown input for these variables. The generation of chemotherapy was based on assumptions (i.e., second-generation chemotherapy). In addition, we did not consider the effects of hormone (or endocrine), trastuzumab and bisphosphonate treatment. The measures aimed to reduce the possibility of a considerable portion of missing data and predictors as they were not typically documented in medical records. We wanted to balance simplicity and the burden of data collection. The survival outcomes after the completion of the extended adjuvant treatment would require a longer cohort follow-up. Despite the limitations, our study showed that a locally adapted myBeST tool was comparable to the western-centric PREDICT tool.

Future works that include these variables and predictors are recommended. They should consist of a cohort with a longer follow-up (i.e., 10 or 15 years) to determine the effects of these predictors on survival in our setting. As a clinical cancer database is being implemented in our country, the database would provide a valuable avenue to derive predictive models for survival among women with breast cancer in Malaysia.

## 5. Conclusions

The PREDICT and myBeST tools had comparable discriminant performance, with myBeST marginally exceed the performance in our sample. Therefore, our locally adapted model may be more suited to convey the survival estimation for women with breast cancer in Malaysia. Nevertheless, considering the drawbacks of both models, they should be used with caution when applied in future practice. Online prediction tools require continuous validation and reproducibility studies to ensure their reliability and usefulness in making a correct intervention strategy.

## Figures and Tables

**Table 1:** Descriptive characteristics of breast cancer patients included in the analysis (n = 532).

Characteristics	n (%)
**Age**, mean (SD) in years	52.1 (10.7)
**Ethnicity**	
Malay	309 (58.1)
Chinese	161 (30.3)
Indian	40 (7.5)
Others	22 (4.1)
**Marital status**	
Married	440 (82.7)
Not married (single/divorced/widowed)	92 (17.3)
**Histological type**	
Ductal carcinoma (NST)	474 (89.1)
Lobular carcinoma	26 (4.9)
Others	32 (6.0)
**Grade**	
Well-differentiated (Grade I)	137 (25.8)
Moderately differentiated (Grade II)	229 (43.0)
Poorly differentiated (Grade III)	166 (31.2)
**ER status**	
Positive	372 (69.9)
Negative	160 (30.1)
**ER and PR status**	
Both ER and PR positive	317 (59.6)
Either ER or PR positive	61 (11.5)
Both negative	154 (28.9)
**HER2 status**	
Positive	140 (26.3)
Negative	332 (62.4)
Unknown	60 (11.3)
**Tumour size**, median (IQR)	29.0 (20.0–45.0)
**Tumour (T) stage**	
T1	133 (25.0)
T2	276 (51.9)
T3	73 (13.7)
T4	50 (9.4)
**Number of positive nodes**, median (IQR)	1.0 (0–2.0)
**Node (N) stage**	
N0	261 (49.1)
N1	186 (35.0)
N2	50 (9.4)
N3	35 (6.6)
**Overall TNM stage**	
I	91 (17.1)
II	285 (53.6)
III	156 (29.3)
**Chemotherapy**	
No	155 (29.1)
Yes	377 (70.9)
**Radiotherapy**	
No	188 (35.3)
Yes	344 (64.7)
**Follow-up time**, median (IQR)	6.1 (5.2–7.5)
**Five-year survival status**	
Alive	427 (80.3)
Dead	105 (19.7)

ER, oestrogen receptor; HER2, human epidermal growth factor receptor 2; IQR, interquartile range; NST, invasive carcinoma of no special type; PR, progesterone receptor; SD, standard deviation; and TNM, tumour node metastasis.

## Data Availability

The data that support the findings are available from the authors, but restrictions apply to the availability of these data. These data were used under agreement for the current study and are not publicly available. Data are, however, available from the authors but only with the explicit permission of the Director General, Ministry of Health Malaysia.
